# Bridging the gap: understanding Belgian anesthesiologists’ proficiency and training demands in gastric point-of-care ultrasound, a case-based survey

**DOI:** 10.1186/s12909-024-05359-5

**Published:** 2024-04-08

**Authors:** Adrien Maseri, Quentin Delhez, Anne-Sophie Dincq, Dominique Lacrosse

**Affiliations:** Anesthesiology Department Centre Hospitalier Universitaire UCL Namur, Site Godinne, Avenue Docteur G. Therasse, 1, 5530 Yvoir, Belgium

**Keywords:** Anesthesiology, Gastrointestinal Contents/diagnostic imaging, Perioperative Care/Methods, Pneumonia, Aspiration/Prevention and Control, Ultrasonography

## Abstract

**Background:**

Pulmonary aspiration syndrome remains a significant complication of general anesthesia, particularly in unfasted patients. Gastric point-of-care ultrasound (POCUS) allows for both qualitative and quantitative assessment of gastric content, providing a safe and reliable method to assess gastric emptying and reduce the risk of aspiration during general anesthesia.

**Methods:**

The survey was distributed to Belgian certified anesthesiologists and trainees between April 2020 and June 2021. Participants received a simulated clinical case of a patient at risk of gastric aspiration, created and approved by two certified anesthesiologists trained to perform gastric POCUS. The objectives of this study were to assess recognition of high-risk clinical situations for gastric aspiration, awareness of the gastric POCUS and its indications, and knowledge of the technical and practical conditions of the procedure among respondents trained in the technique. Furthermore, the study assessed the state of training in gastric POCUS, the desire for education, and the practical availability of ultrasound equipment. The survey used conditional branching to ensure unbiased responses to POCUS-related questions. It included multiple-choice questions, quantitative variables, and 5-point Likert scales. The margin of error was calculated using Daniel’s formula, corrected for a finite population.

**Results:**

The survey was conducted among 323 anesthesiologists. Only 20.8% (27) recognized the risk of a full stomach based on the patient’s history. Anesthesiologists who recognized the indication for gastric POCUS and were trained in the procedure demonstrated good recall of the practical conditions for performing the procedure and interpreting the results. Only 13.08% (31) of all respondents had received training in gastric POCUS, while 72.57% (172) expressed interest in future training. Furthermore, 80.17% (190) of participants had access to adequate ultrasound equipment and 78.90% (187) supported teaching gastric POCUS to anesthesia trainees.

**Conclusions:**

This survey offers insight into the epidemiology, clinical recognition, knowledge, and utilization of gastric POCUS among Belgian anesthesia professionals. The results emphasize the significance of proper equipment and training to ensure the safe and effective implementation of gastric POCUS in anesthesia practice. Additional efforts should focus on improving training and promoting the integration of gastric POCUS into daily clinical practice.

**Supplementary Information:**

The online version contains supplementary material available at 10.1186/s12909-024-05359-5.

## Background

Pulmonary aspiration of gastric contents is a serious complication of general anesthesia, with a prevalence ranging from 1:3000 to 1:6000 in elective surgery and 1:600 in emergency surgery in adults [1]. It is also associated with a mortality rate of up to 27.8% [2].

To prevent the flow of stomach contents into the upper airway, it is crucial to ensure gastric emptying by allowing sufficient fasting time. However, various factors such as failure to fast, bowel obstruction, pregnancy, pathologic delayed gastric emptying [3], or medications such as GLP1 agonists [4] may render this time insufficient. Chronic respiratory failure can also be a lesser known risk factor, probably due to pathologic changes associated with chronic hypoxemia, COPD therapy, and autonomic dysregulation [5–7].

Since 1980, gastric ultrasound has been developed as a noninvasive method to assess gastric emptying [8]. This technique was refined by teams led by Bouvet, Perlas, and Van de Putte [9–12], resulting in a common technique called gastric point-of-care ultrasound (gastric POCUS). This technique is highly effective and reliable in assessing gastric content and volume [13].

The method allows for both qualitative (solid, liquid, or empty) and quantitative assessment of gastric filling status [14]. To measure the cross-section of the antral surface, a 3.5-5 MHz curved ultrasound probe is used while the patient is in the supine position and then in the right lateral decubitus position for fluid analysis. The left lobe of the liver, pancreas, abdominal aorta, and superior mesenteric artery serve as anatomical landmarks for precise measurement of this area. This section provides a method for calculating the gastric residual volume based on the patient’s age, with a recommended limit of less than 1.5 ml/kg [12].

The Perlas grading system [15] is a reliable method for qualitative assessment. This system categorizes patients into three distinct grades based on the amount of fluid detectable in the antrum: Grade 0 (empty antrum), Grade 1 (minimal fluid detectable only in the right lateral decubitus position), and Grade 2 (markedly distended antrum with visible fluid in both supine and lateral positions).

The incorporation of Point-of-Care Ultrasound (POCUS), including Gastric POCUS, into anesthesiologist basic training has gained international support. Canada [16] and the United States [17] have already integrated POCUS education, with a specific emphasis on Gastric POCUS, into their core anesthesiology training programs. In 2020, a panel of experts in Belgium convened to develop recommendations for integrating Gastric POCUS into the basic training curriculum for anesthesiologists [18]. The focus was on proposing a comprehensive framework for seamlessly integrating this technique into educational pathways. Although initiatives have been taken, current research in Belgium has not yet explored the extent of interest among anesthesiologists regarding Gastric POCUS training and its integration into formal educational programs.

This study examines the clinical recognition of high-risk situations associated with a full stomach, awareness of gastric POCUS, understanding of the technical and practical aspects of the technique, and its potential impact on the induction plan.

## Materials and methods

To address the aforementioned areas, we created a conditional branching survey that enables participants to be directed to specific sections based on their previous responses. Please refer to Table 1 for the questionnaire items and their corresponding conditional branches.

**Table 1:** Questionnaire creation process based on clinical questions

DIMENSIONS	COMPONENTS	QUESTIONS
Epidemiology Q1 - Q5	Age, gender, level of training, type of practice	
Decoy Question Q6	How do you perform anesthesia for colonoscopy in your establishment?	
Clinical recognition of ‘at-risk’ situation Q7 - Q8	Actions to be taken if a “high risk” full stomach situation is suspected (e.g. gastric US, gastroscopy, CT scan, straightforward planning of rapid sequence induction).	Do you have enough information to induce anesthesia?
		What is your plan to induce anesthesia?
Decoy questions Q9– Q11	What drugs will you use to induce anesthesia About the maintenance of anesthesia (Inhaled, Target controlled infusion) Where does the patient go after the colonoscopy? (recovery room, ICU, home)	
Knowledge of the existence of the technique Q12	Knowledge of the existence of the technique and its accessibility to the anesthesiologist	In this situation, do you propose to proceed to Gastric-POCUS?
Realization of the technique Q13	Knowledge of technical aspects	What kind of US-probe do you use?
	Knowledge of patient positioning aspect	In which position do you proceed to the examination?
	Knowledge of anatomical landmarks	What are the necessary anatomical landmarks to obtain an interpretable image?
Interpreting the results of Gastric POCUS Q14 - Q15	Qualitative analysis of the result	Do you detect ‘at-risk’ gastric content?
	Qualitative analysis of the result	Given the result of liquid volume evaluation, is the situation ‘at-risk’?
Clinical decision according to the result Q16– Q19	Clinical decision on anesthesia plan based on gastric content evaluation	Do you delay the intervention?
		If it is not possible to reschedule the patient, how do you proceed with the induction of anesthesia?
Field conditions for performing a Gastric-POCUS Q20– Q22	State of training in Gastric POCUS	Are you trained in performing Gastric-POCUS and, if not, do you want to?
	Availability of the correct US-probe	Do you have a low-frequency US-probe available?
	Training of future specialists	Should Gastric-POCUS be more present in basic anesthesiology training?

### Questionnaire and variables

The study targeted all clinically active anesthesiologists in Belgium, including 3023 certified anesthesiologists and 638 anesthesiology trainees, as reported by the National Institute for Health and Disability Insurance (NIHDI) [19]. Participation in the survey was voluntary, and respondents were not compensated. No participants were excluded based on any criteria. By completing the questionnaire, participants provided their consent for the analysis and use of their responses.

The survey sample size was confidently determined using the corrected Daniel formula for a finite population of 3661 anesthesiologists in Belgium. A confidence level of 95% and an acceptable margin of error of 0.05 were assertively considered, resulting in a formula-calculated requirement of 348 participants for the study, assuming a sample proportion of 0.50.

A cross-sectional survey was administered on a computer-based platform with a total of 22 questions. It included multiple-choice questions (MCQs), quantitative variable questions, and Likert scale questions. The text adhered to conventional academic structure and style guidelines. It avoided biased, emotional, or ornamental language and opted for clear, objective, and value-neutral language with a passive tone and impersonal construction. The writing maintained a formal register and avoided contractions, colloquial words, informal expressions, and unnecessary jargon. The structure was clear and progressive, and the writing was free from grammatical errors, spelling mistakes, and punctuation errors. Technical terms were used consistently throughout the text, and abbreviations were explained upon first usage. The survey was designed with a non-linear structure to improve data accuracy, requiring respondents to answer all questions without revisiting previous answers. Additionally, conditional branching was incorporated to prevent specific questions related to Gastric POCUS from being imposed on respondents who primarily rely on clinical judgment to induce anesthesia. Additionally, technical questions about gastric POCUS were directed only to those who had received formal training. Table 1 presents a comprehensive diagram of the survey’s sequential process.

The initial questions (Q1-Q6) gathered epidemiological data such as gender, age, education level, practice type, and clinical practice location. Question 7 assessed participants’ ability to identify situations with a high risk of a full stomach by presenting a clinical scenario of a patient undergoing colonoscopy prior to lung transplantation [see Additional file 1]. Question 12 evaluated participants’ interest in obtaining additional information about the case and their desire to perform gastric POCUS while determining their training status. Trained participants then answered questions 13–15, which assessed the technical and anatomical prerequisites for performing gastric POCUS.

Additionally, an image of gastric POCUS was presented to the participants (Q16-Q19), which provided information on the ultrasound-derived gastric volume measurements. The survey inquired about the participants’ anesthesia induction method, the availability of ultrasound equipment, and their views on incorporating POCUS training into the academic curriculum for anesthesia trainees. Please see [Additional file 1] for a comprehensive survey content.

The authors confidently pretested the survey at their institution and collected 11 responses. The primary objective was to confidently identify potential problems with understanding the questionnaire. The sample included certified anesthesiologists, some of whom practiced exclusively in clinical settings, while others were also involved in academic research.

### Data collection

The SurveyMonkey® survey was electronically distributed to active certified anesthesiologists and trainees working in hospitals throughout Belgium from April 2020 to June 2021. To target this specific group, a comprehensive list of surgical hospitals compiled by the FPS Public Health [20] was used. A total of 117 anesthesia departments were contacted either through a contact form or direct mail to the anesthesia department secretary.

To increase the reach and participation of the survey, it was also made easily accessible on the authoritative websites of renowned professional associations, specifically the Belgian Society of Anesthesiology, Intensive Care, Perioperative Medicine and Pain Management and the Belgian Association for Regional Anesthesia. The survey aimed to gather a diverse and inclusive group of anesthesiologists from all regions of Belgium by utilizing established digital platforms.

The email distributing the survey clearly stated that individuals who had already completed it should not participate. This was done to prevent duplicate responses from those who may have already participated or received the survey through other channels. By prohibiting multiple responses, the goal was to collect distinct and unique feedback from each participant.

### Statistical analysis

Demographic characteristics, such as age (in years), sex (male/female), credentials (certified/trainee), practice type (university/public/private/mixed), and region, were analyzed using descriptive statistics. Numerical variables were presented as means and standard deviations, while categorical variables were presented as percentages. The same method was used to summarize categorical variables in the rest of the survey.

The survey used a Likert scale to measure respondents’ agreement or disagreement, ranging from 1 (strongly disagree) to 5 (strongly agree). The weighted Likert score (WLS) was calculated by averaging the responses. This method enables a comprehensive understanding of the level of agreement or disagreement among respondents. However, detailed analysis of individual responses is not possible with this approach. To offer a more complete analysis, the numerical summary is complemented by a graphical representation that displays the distribution of responses.

## Results

The survey study received 323 responses, with 223 from certified anesthesiologists and 100 from trainees. To calculate the final margin of error, we used the Daniel formula corrected for a finite population, resulting in a margin of error of 5.21%. This margin of error was then used throughout the remainder of the article.

### Participant flow

**Fig. 1:**
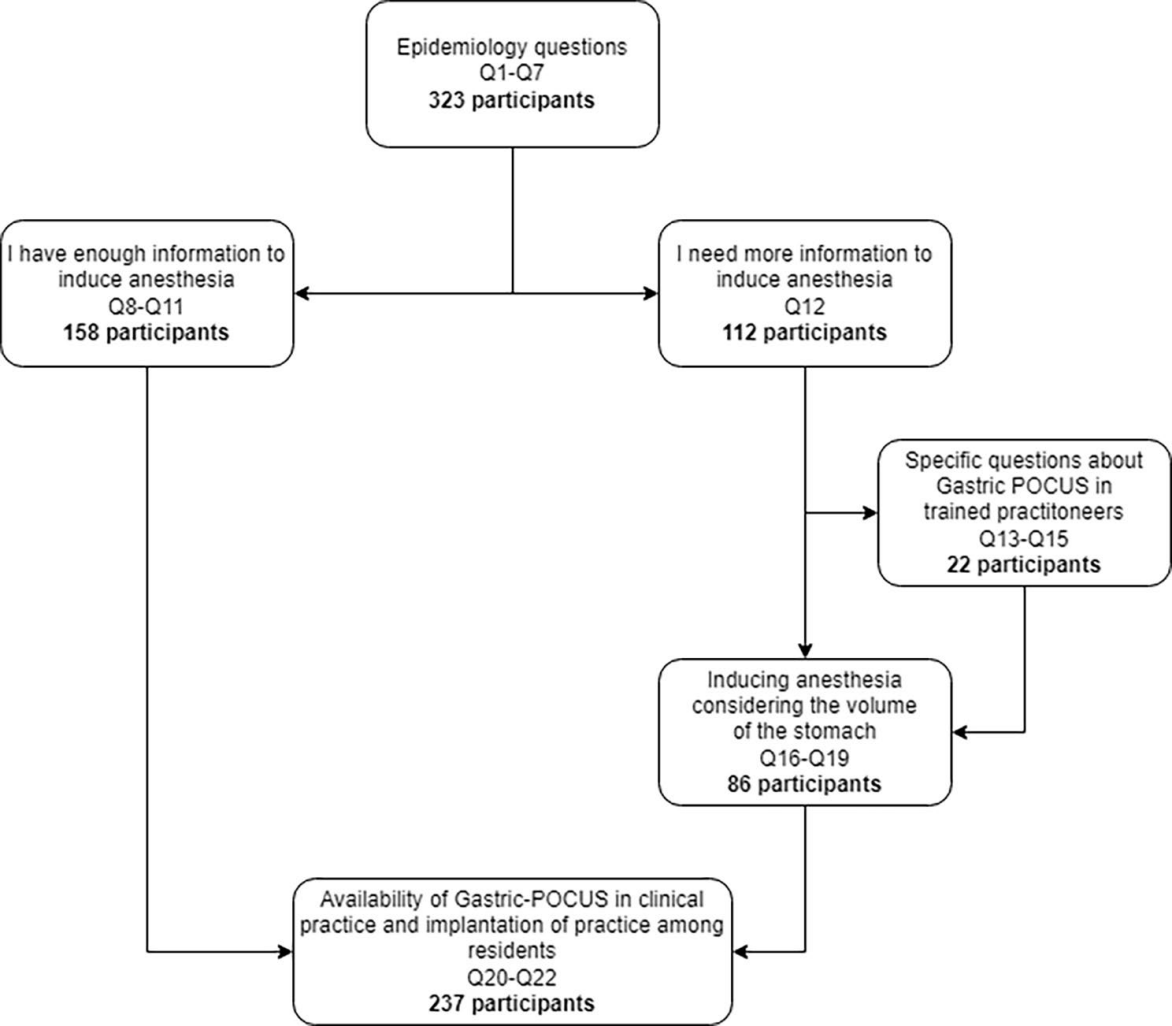
Flowchart for participant distribution across survey Sect. 8

A flowchart of the participant distribution across the survey section is shown in Fig. 1.

Among the respondents, 170 individuals chose direct induction of anesthesia. Of these, 158 participants continued the survey and specified their chosen induction type. Furthermore, 112 respondents requested additional information about the clinical case, with 111 providing specific details of their inquiries. Among them, 22 respondents had prior training in gastric POCUS and were asked about its practical performance and interpretation. Finally, 237 respondents completed the survey, indicating their training preferences and whether they had the required ultrasound equipment available.

### Descriptive results

The survey had a final completion rate of 69%, with an average completion time of 4 min and 39 s. To comply with the General Data Protection Regulation (GDPR) and ensure anonymity, only the IP addresses of the participants were retained by the computer system. No matches were found when pooling responses and IP addresses. The reappearance of certain IP addresses may be due to the use of shared computers in different anesthesia departments. However, there is no guarantee that the same person did not respond twice from two different IP addresses.

### Main findings

#### Question 1 to 5: epidemiology

The sociodemographic characteristics of the participants are described in Table 2.

**Table 2:** Sociodemographic of participants

	Certified Anesthesiologist (*n* = 223)		Anesthesiology trainee (*n* = 100)		Total (*n* = 323)	
Gender						
Male	133	59.64%	48	48.00%	181	56.04%
Female	89	39.91%	50	50.00%	139	43.03%
Other	1	0.45%	2	2.00%	3	0.93%
Mean age (years ± SD)						
Total	45 ± 10		30 ± 6		40 ± 11	
Type of practice						
University hospital	58	26.01%	67	67.00%	125	38.70%
Public hospital	86	38.57%	19	19.00%	105	32.51%
Private hospital	66	29.60%	9	9.00%	75	23.22%
Mixed Practice	13	5.83%	5	5.00%	18	5.57%

### Question 7 for clinical recognition of “At-risk” Situation

The clinical recognition of an “at-risk” situation is detailed in Table 3.

**Table 3:** Clinical recognition of “at-risk” situation

	Direct induction of anesthesia (*n* = 170)			Needing more information (*n* = 113)	
Rapid sequence induction and intubation	21	13.29%	Gastric POCUS	27	24.11%
Other types of induction	137	86.71%	Other Gastric evaluation	19	16.96%
			Other information needed	66	58.93%
Discontinuation of survey	12	/	Discontinuation of survey	1	0.89%

Out of the 323 responses obtained, 170 participants chose direct induction of anesthesia. Among them, 158 specified the type of induction used. Rapid sequence induction and intubation (RSII) was chosen by 13.29% (*n* = 21) of the participants. Furthermore, among the 112 individuals who requested further testing, 41.07% (*n* = 46) inquired about gastric emptying, while 24.11% (*n* = 27) specifically requested gastric point-of-care ultrasound (POCUS). Overall, only 25.81% (*n* = 67) of participants identified the risk of a full stomach based on clinical symptoms and patient risk factors.

### Question 12: knowledge of the indications for gastric POCUS

Of the 111 participants who responded to the Q12 survey on fasting assessment, 85.6% (*n* = 95) recognized the indication for gastric ultrasound. Of these, 25.2% (*n* = 28) reported having received training in gastric ultrasound and specifically recognized the indication, while 60.4% (*n* = 67) reported recognizing the indication despite having no specific training in this area. Despite this, 14.4% (*n* = 16) of participants stated that gastric ultrasound was not necessary.

### Question 13: realization of the technique

**Fig. 2:**
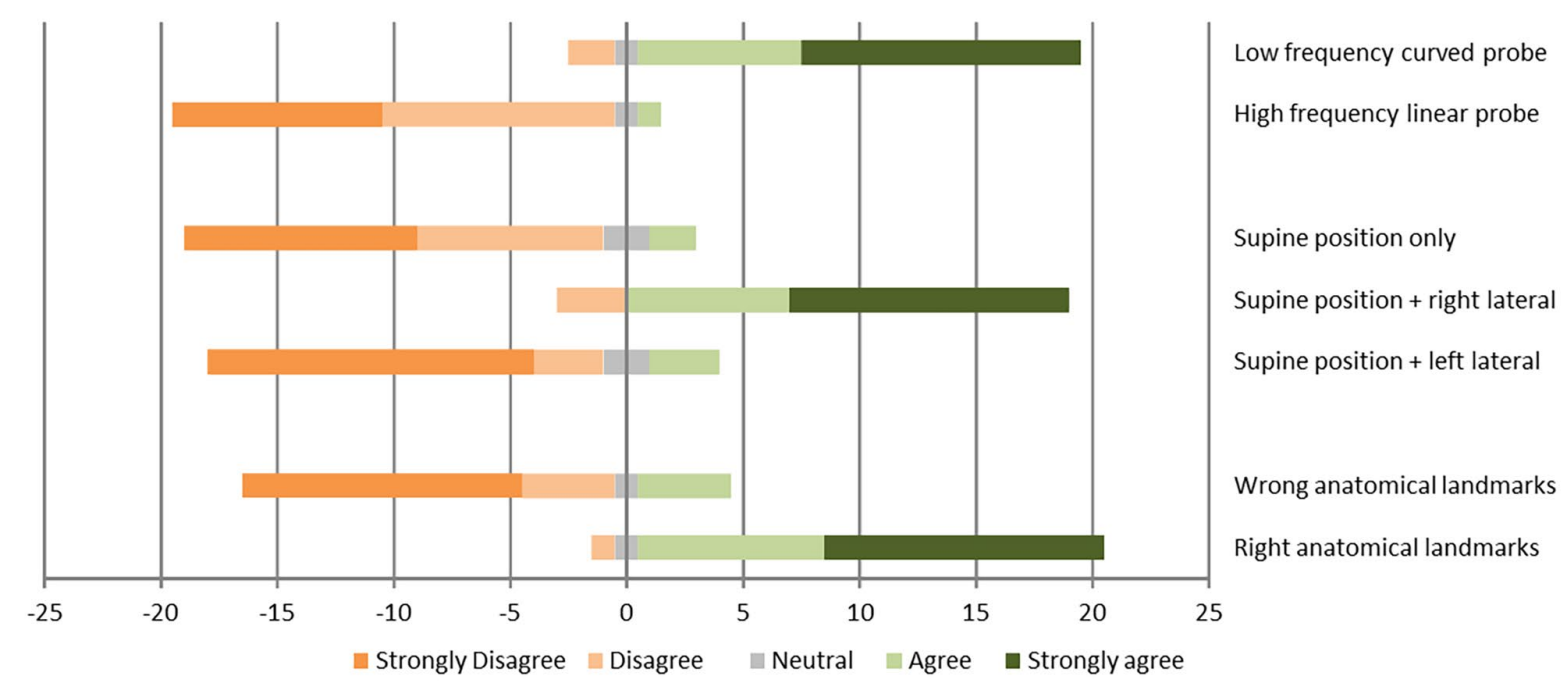
Representation of Likert scale responses regarding the clinical context of performing gastric point-of-care ultrasound (POCUS) 10

The following results were obtained from the responses of the 22 trained participants. Our study used a WLS ranging from 1 to 5, and the results are shown in Fig. 2.

### Technical aspects

A mean WLS of 4.32 was observed for the use of the low-frequency curvilinear probe, indicating strong overall agreement with its use. In contrast, the high-frequency linear probe yielded a mean WLS of 1.71 and is perceived to have limited effectiveness in adult subjects.

### Patient positioning aspect

Examination in the supine position followed by the right lateral decubitus position yielded a mean WLS of 4.27, suggesting strong overall agreement with this approach. The other positions showed strong overall disagreement, with a mean WLS of 1.82 for the dorsal position only and a mean WLS of 1.73 for the supine position followed by the left lateral decubitus position.

### Anatomical landmarks

Using anatomic landmarks such as the left hepatic lobe, pancreas, abdominal aorta, and superior mesenteric artery to divide the gastric antrum resulted in a mean WLS of 4.41, demonstrating strong overall agreement with this approach. However, the use of anatomic landmarks such as the left hepatic lobe, pancreas, inferior vena cava, and left renal vein resulted in a mean WLS of 1.86, indicating overall robustness.

### Questions 14 and 15: interpreting the results of gastric POCUS

#### Qualitative analysis of the results

Among respondents, 63.64% (*n* = 14) identified the stomach as full based on the image provided and the question title. However, 31.82% (*n* = 7) of participants required additional information and chose to measure the antral surface to accurately assess gastric fullness, while 4.6% (*n* = 1) considered the stomach empty. Notably, among those seeking further details, 57.1% (*n* = 4) specifically requested an antral surface measurement or Perlas grading system assessment. Upon receiving additional information, participants were apprised of the measured gastric fluid volume, which totaled 3.3 ml/kg, indicative of a high-risk scenario surpassing the 1.5 ml/kg threshold of an empty stomach.

### Question 16 to 19: clinical decision according to POCUS

The majority of respondents were in favor of delaying the procedure until later in the day (mean WLS 3.91). However, they were less inclined to delay the procedure until the next day (mean WLS 2.30). If the procedure could not be postponed, 70.93% (*n* = 61) of the respondents opted for rapid RSII, while 11.63% (*n* = 10) opted for general anesthesia with endotracheal intubation without RSII. A total of 1.16% (*n* = 1) preferred the use of a laryngeal mask, and 16.7% (*n* = 14) performed procedural sedation despite the risk of inhalation.

### Questions 20 to 22: field conditions for performing a gastric POCUS

A total of 237 respondents answered the questions in this section, for a margin of error of 6.16%. The results are shown in Table 4.

**Table 4:** Field conditions for performing a Gastric-POCUS

	Certified Anesthesiologist (*n* = 172)		Anesthesiology trainee (*n* = 65)		Total (*n* = 237)	
State of training in Gastric POCUS						
Trained	26	15.12%	5	7.69%	31	13.08%
Desire to be trained	117	68.02%	55	84.62%	172	72.57%
Untrained and do not want to be	29	16.86%	5	7.69%	34	14.35%
Availability of abdominal US probe						
Yes	139	80.81%	51	78.46%	190	80.17%
No	24	13.95%	2	3.08%	26	10.97%
Unknown	9	5.23%	12	18.46%	21	8.86%
Gastric Point-of-care Ultrasound should be more present in basic anesthesiology training						
Yes	128	74.42%	59	90.77%	187	78.9%
No	9	5.23%	1	1.54%	10	4.22%
Neutral	35	20.35%	5	7.69%	40	16.88%

### State of training in gastric POCUS

Among the respondents, 13.08% (*n* = 31) had been trained in gastric POCUS. Conversely, 72.57% (*n* = 172) of the respondents were not trained but would like to be trained, while 14.35% (*n* = 34) were not trained and did not want to be trained.

#### Availability of the correct US probe

Regarding the availability of the appropriate ultrasound probe, 80.17% (*n* = 190) of participants had access to a low-frequency curved ultrasound probe. However, 10.97% (*n* = 26) of participants did not have this probe, and 8.86% (*n* = 21) were unsure of its availability.

### Training of future specialists

Regarding the training of future specialists, a majority of the 78.90% (*n* = 187) of respondents were in favor of teaching gastric POCUS to trainees, while only 4.22% (*n* = 10) were opposed and 16.88% (*n* = 40) were neutral.

## Discussion

The survey study received 323 responses from 223 certified anesthesiologists and 100 trainees. Certified anesthesiologists had an average age of 45 years, while trainees had an average age of 30 years. Practice settings were evenly distributed among university-affiliated, public, and private practices. Notably, only 26% of respondents identified the risk of a full stomach. Respondents trained in gastric POCUS demonstrated a proficient understanding of the technical requirements and interpretation of gastric POCUS, along with its implications for general anesthesia induction. It is worth noting that demand for gastric POCUS training was high among both certified anesthesiologists and trainees, and the majority of anesthesia services were equipped with abdominal ultrasound probes. A significant number of certified anesthesiologists and trainees support the inclusion of gastric POCUS in the basic anesthesia training curriculum.

### Interpretations

### Clinical recognition of a “high risk” situation for the full stomach

Our study found that only 20.8% of respondents were able to detect a full stomach situation. This was identified through rapid sequence induction or additional examination targeting gastric filling status. Surprisingly, frequent nausea and early satiety, which may indicate gastric fullness, appear to be clinically under-recognized by the respondents. This study emphasizes the significance of increased awareness and education on clinical signs, as prompt identification of gastric fullness is essential for patient safety and perioperative management. Additionally, the results suggest that chronic respiratory insufficiency is a significantly under-recognized risk factor among anesthesiologists. Efforts to improve clinicians’ awareness and understanding of these important but subtle symptoms are warranted to improve patient outcomes and reduce perioperative complications.

### Indications, technical aspects and interpretations of gastric POCUS

The vast majority of respondents who suspected delayed gastric emptying recognized the relevance of gastric POCUS in our clinical situation. However, only 13.1% of respondents had received prior training in the technique.

Among the trained individuals, there was unanimous agreement on the use of a low-frequency curved ultrasound probe and the supine position followed by lateral testing, indicating a clear understanding of the technical standards for performing gastric POCUS. The participants demonstrated a high level of proficiency in identifying the necessary anatomical landmarks required for performing the technique. Nearly all respondents were able to identify the presence of fluid in the stomach while interpreting the ultrasound image. Additionally, approximately one-third of the participants requested that the antral cross-sectional area be measured to determine whether the fluid was abnormal. A considerable number of participants reported abnormal fluid volume based on images without measurements, which may be related to the large amount of fluid present in the image. An experienced echographer may be able to estimate the gastric volume based on their experience.

It was generally agreed that the procedure should be postponed until later in the day, but not until the following day. When faced with the need to proceed quickly, most respondents with gastric ultrasound training either performed RSII or administered general anesthesia with intubation. Despite the disclosure of a volume of gastric fluid that exceeded the high-risk threshold by more than twofold, 17% of respondents still administered procedural sedation without securing the airway.

### State of training and environmental conditions in gastric POCUS

Few respondents have received training in performing gastric POCUS, yet there is a high demand for training, particularly among trainees. Additionally, most users have access to adequate ultrasound equipment. 79% of respondents were convinced that Gastric POCUS should be more present in future specialists’ training.

The American Society of Anesthesiologists [14, 17] has developed a certifying program in point-of-care ultrasound (Diagnostic POCUS Certificate Program) following the integration of POCUS ultrasound examinations into the APPLIED Exam Objective Structured Clinical Examination (OSCE) by the American Board of Anesthesiology. There are also recommendations from the European Union of Medical Specialists regarding Gastric POCUS education [21]. Training courses are currently being developed and delivered at both European and Belgian levels by the European Society of Anesthesia and Intensive Care (EUROPoCUS program) and UZLeuven (BePOCUS program), respectively.

The Belgian Society of Anesthesiology, Resuscitation, Perioperative Medicine and Pain Management (BeSARPP) has raised concerns about the implementation of training due to the medico-legal implications of certification in Gastric POCUS [21]. Nevertheless, they are willing to be involved in this project, in accordance with their mission to promote and improve education in perioperative medicine.

### Limitations

Although the calculated sample size was initially set at 348 respondents to achieve a 5% error margin, only 323 responses were received. To address this discrepancy, we used the corrected Daniel formula for a finite population and applied reverse calculation, resulting in a final margin of error of 5.21%. Although the response rate among certified anesthesiologists may raise concerns about representativeness, we conducted a subgroup analysis to assess the issue. The subgroup calculation resulted in a final margin of error of 6.32% based on the 223 responses out of 3023 active certified anesthesiologists. The interpretation of the results remains robust despite this slightly increased margin of error due to the strong and conclusive findings obtained.

In Belgium, Dutch and French are the two predominant languages. To prevent potential translation errors, the authors chose to use English. However, some practitioners may find this discouraging, which could limit the response rate. Additionally, the number of survey dropouts may be due to the survey’s potential length, despite the mean completion time being 4 min and 39 s.

There may be other potential influences to take into account, such as the potential effect of frequent mailbox congestion on the visibility of the electronic survey or on participants’ desire to engage. However, using professional email addresses and working with anesthesia departments can help to mitigate this possibility of bias. Despite the measures implemented at the outset of the study, it is not possible to guarantee that no participant completed the survey more than once. It is important to consider the potential consequences of study dropouts, as they may lead to greater imprecision of responses as the questionnaire progresses. The margin of error percentage had to be revised upward as the survey progressed. Inaccuracies could also have been created due to survey question design and the inclusion of decoy questions to avoid response bias on our research topic.

### Generalizability

The generalization of the study’s findings may be limited by several factors. Firstly, Belgium operates within a distinct educational framework that differs from neighboring countries. The intricacies of Belgium’s training system, coupled with regional disparities and legislative nuances, may impede the direct extrapolation of the results to other nations. Therefore, while our study provides valuable insights into the perceptions and practices of anesthesiologists in Belgium, it is important to exercise caution when attempting to generalize these findings to healthcare settings in other countries.

## Conclusions

Our survey-based study provides insight into the clinical practice and educational needs of certified anesthesiologists and trainees in Belgium regarding gastric POCUS. The study highlights a concerning lack of clinical recognition among Belgian anesthesiologists regarding the risk of pulmonary aspiration. It is worth noting that although most respondents who suspected gastric fullness recognized the indication for gastric point-of-care ultrasound (POCUS), very few had received formal training in its execution. Proficiency in performing and interpreting gastric POCUS examinations appears to be well integrated among those who have been trained in the technique. Additionally, our findings indicate that the required equipment to perform gastric POCUS is readily available in the majority of anesthesia services surveyed. The strong interest in training for gastric POCUS techniques, expressed by both trainees and certified professionals, emphasizes its perceived importance in clinical practice. Overall, the respondents support the implementation of gastric POCUS training as a basic component of anesthesia training, highlighting its potential benefits. These insights offer valuable guidance for developing curricula and professional training initiatives to improve patient safety and procedural competency in anesthesia practice.

### Electronic supplementary material

Below is the link to the electronic supplementary material.

Supplementary Material 1

## Data Availability

The data that support the findings of this study are not openly available due to the presence of personal information and the nature of the survey related to practice quality. Data is available from the corresponding author upon reasonable request. Data are located in controlled access data storage at CHU UCL Namur.

## Abbreviations

NIHDI: National Institute for Health and Disability Insurance
POCUS: Point-of-care ultrasound
RSII: Rapid sequence induction and intubation
WLS: Weighted Likert scale
