# Supplementary material for: Bridging the gap: understanding Belgian anesthesiologists’ proficiency and training demands in gastric point-of-care ultrasound, a case-based survey
Source: BMC Med Educ. 2024 Apr 8;24:383. doi: 10.1186/s12909-024-05359-5 (PMC11003122; doi:10.1186/s12909-024-05359-5)
Supplement: Supplementary file 1 — Supplementary Material 1 [file 12909_2024_5359_MOESM1_ESM.pdf]

CHU UCL NAMUR

Participant information

*** 1. Gender**

- ◯ Male
- ◯ Female
- ◯ Other

*** 2. How old are you ?**

*** 3. Your actual level of training is...**

- ◯ Senior Anesthesiologist
- ◯ Resident Anesthesiologist

*** 4. What kind of hospital are you working for ?**

- ◯ University hospital
- ◯ Public hospital
- ◯ Private hospital
- ◯ Mixed practice

*** 5. In which Province do you work ?**

- ◯ Antwerpen
- ◯ Brabant Wallon
- ◯ Brussels
- ◯ Hainaut
- ◯ Liège
- ◯ Limburg
- ◯ Luxembourg
- ◯ Namur
- ◯ Oost-Vlaanderen
- ◯ Vlaams Brabant
- ◯ West-Vlaanderen

*** 6. How do you perform anesthesia for colonoscopy in your establishment ?**

	never	rarely	frequently	always
Colonoscopies are performed in the OR	○	○	○	○
Colonoscopies are performed in day-surgery service	○	○	○	○
Colonoscopies are performed in an independent room from OR/Day-surgery	○	○	○	○
Patients have a scheduled preoperative assessment	○	○	○	○
Patients do not systematically have a preoperative assessment but a form is sent several days before colonoscopy	○	○	○	○

Clinical Case

Today 8 a.m., you are taking care of a 50 years old man, 55kg, B.M.I. 18. He must undergo a colonoscopy in the setting of a pre-pulmonary transplant health check.

His medical history includes:

- Chronic respiratory failure (COPD GOLD IV), depending on oxygen 2 l/min 24h/24h
- No ischemic heart disease, no pulmonary hypertension, no valvular disease
- No gastroesophagal reflux
- No diabetes
- No morphinic nor tricyclic antidepressants are part of his medication

The last time he ate was 8pm yesterday and he finished to drink his colic preparation at 5.30 am. During the preoperative checkup, the patient reported digestive complaints as nausea and early satiety. The patient has also lost weight.

*** 7. Do you have enough informations to induce anesthesia ?**

- ◯ I have enough informations
- ◯ I need additional tests before inducing anesthesia. Please specifies which ones:

Anesthesia Induction

*** 8. What is your plan of anesthesia ?**

- ◯ Procedural sedation with nasal oxygen
- ◯ Procedural sedation with facemask oxygen
- ◯ General anesthesia with Supraglottic Airway Device
- ◯ General anesthesia with Tracheal intubation
- ◯ Rapid sequence induction and intubation (RSII)

*** 9. What drugs will you use to induce anesthesia ?**

- ☐ Midazolam
- ☐ Propofol
- ☐ Ketamine
- ☐ Etomidate
- ☐ Fentanyl
- ☐ Sufentanil
- ☐ Rocuronium
- ☐ Atracurium
- ☐ Suxamethonium

*** 10. About the maintenance of anesthesia**

- ◯ Continuous infusion of propofol
- ◯ Target Controlled Infusion of propofol
- ◯ Sevoflurane
- ◯ Desflurane

*** 11. After the coloscopy :**

	strongly disagree	disagree	neutral	agree	strongly agree
The patient directely goes back to his room	○	○	○	○	○
The patient goes in a recovery room	○	○	○	○	○
the patient goes to the ICU	○	○	○	○	○
The patient goes home on the same day	○	○	○	○	○
The patient stays in the hospital for one night	○	○	○	○	○

Pre-anesthetic use of Gastric Ultrasound

Today 8 a.m., you are taking care of a 50 years old man, 55kg, B.M.I. 18. He must undergo a colonoscopy in the setting of a pre-pulmonary transplant health check.

His medical history includes:

- Chronic respiratory failure (COPD GOLD IV), depending on oxygen 2 l/min 24h/24h
- No ischemic heart disease, no pulmonary hypertension, no valvular disease
- No gastroesophagal reflux
- No diabetes
- No morphinic nor tricyclic antidepressants are part of his medication

The last time he ate was 8pm yesterday and he finished to drink his colic preparation at 5.30 am. During the preoperative checkup, the patient reported digestive complaints as nausea and early satiety. The patient has also lost weight.

*** 12. Concerning fasting evaluation, in this case...**

- ◯ I perform a Gastric Point of Care Ultrasound
- ◯ A Gastric Point of Care Ultrasound is indicated but I am not trained to do it
- ◯ A Gastric Point of Care Ultrasound is not indicated

Ultrasonography results

You proceed to the ultrasound exam in the right lateral position obtaining the following result :

Gastric ultrasound imaging

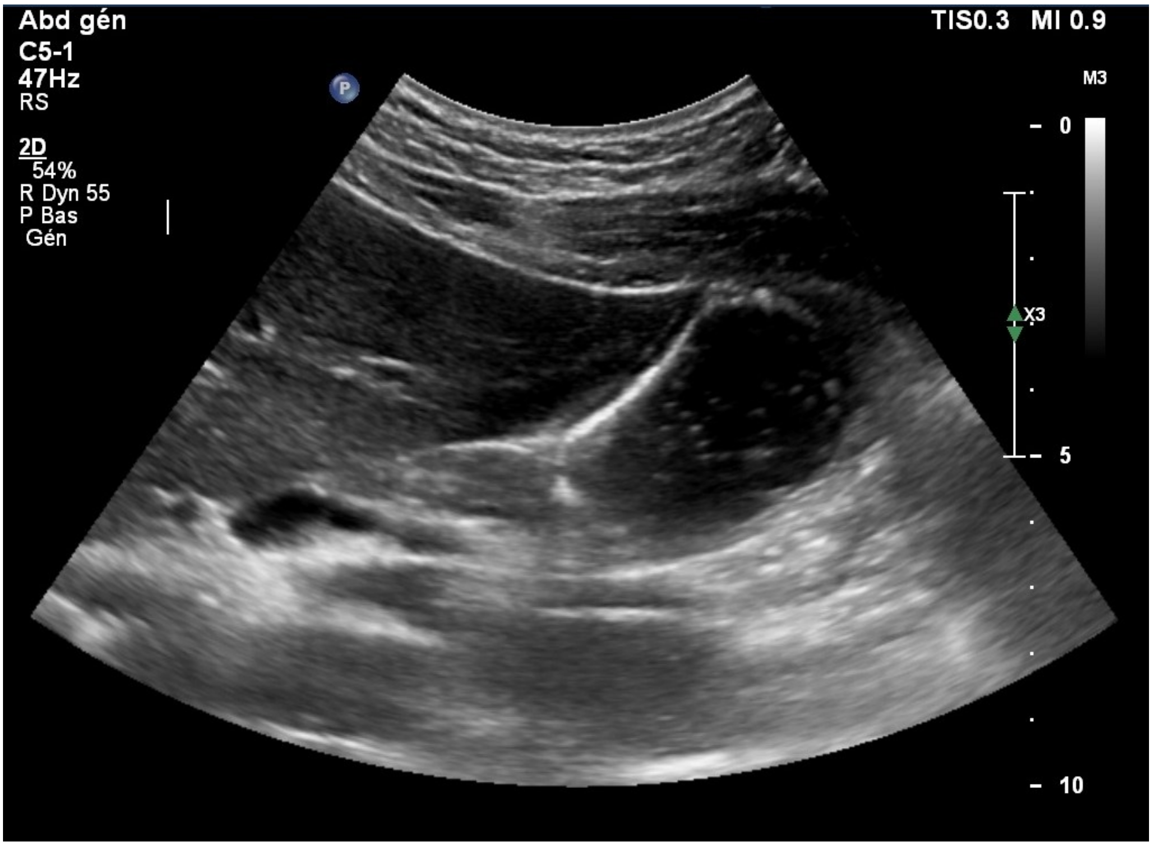

*** 13. Concerning the ultrasound settings to perform a Gastric POCUS for this patient...**

	strongly disagree	disagree	neutral	agree	strongly agree
I use a low frequency curved probe	◯	◯	◯	◯	◯
I use a high frequency linear probe	◯	◯	◯	◯	◯
I perform the test with the patient in supine position only	◯	◯	◯	◯	◯
I perform the test with the pation in supine position then in right lateral postion	◯	◯	◯	◯	◯
I perform the test with the pation in supine position then in left lateral postion	◯	◯	◯	◯	◯
I perform an echographic slice of gastric fundus. My anatomical landmarks are : left liver lobe, pancreas, inferior vena cava and left renal vein	◯	◯	◯	◯	◯
I realize an echographic slice of gastric antrum. My anatomical landmarks are : left liver lobe, pancreas, abdominal aorta and superior mesenteric artery	◯	◯	◯	◯	◯

*** 14. Based on the image above...**

- ◯ I have enough information to affirm the stomach is empty
- ◯ I have enough information to affirm the stomach is full
- ◯ I don't have enough information to affirm the fasting status of the patient

15. If you cannot decide, please tell us what other information you need.

Definitive anesthetic management

Your measurements of the cross-section of the antrum is 15 cm² corresponding to a volume of 182 ml (3,3 ml/kg)

*** 16. Concernig anesthetic induction :**

	strongly disagree	disagree	neutral	agree	strongly agree
I perform anesthesia on a stretcher	○	○	○	○	○
I perform anesthesia on a classic OR table	○	○	○	○	○
I perform anesthesia later that day	○	○	○	○	○
There is no anesthesia, the patient is rescheduled another day	○	○	○	○	○

*** 17. It is impossible to delay the procedure and you decide to induce anesthesia. What do you suggest?**

- ○ Procedural sedation with nasal oxygen
- ○ Procedural sedation with facemask oxygen
- ○ General anesthesia with Supraglottic Airway Device
- ○ General anesthesia with Tracheal intubation
- ○ Rapid sequence induction and intubation (RSII)

*** 18. What drugs will you use to induce anesthesia ?**

- [ ] Midazolam
- [ ] Propofol
- [ ] Ketamine
- [ ] Etomidate
- [ ] Fentanyl
- [ ] Sufentanil
- [ ] Rocuronium
- [ ] Atracurium
- [ ] Suxamethonium

*** 19. After the coloscopy :**

	strongly disagree	disagree	neutral	agree	strongly agree
The patient directely goes back to his room	○	○	○	○	○
The patient goes in a recovery room	○	○	○	○	○
the patient goes to the ICU	○	○	○	○	○
The patient goes home on the same day	○	○	○	○	○
The patient stays in the hospital for one night	○	○	○	○	○

Clinical usage of Gastric Ultrasound

*** 20. About Gastric Point-of-care Ultrasound...**

- ◯ I am actually trained in performing Gastric POCUS
- ◯ I am not trained to perform Gastric POCUS but I would like to
- ◯ I am not trained to perform Gastric POCUS and I do not want to

*** 21. In my anaesthesia department...**

- ◯ We have a curved low-frequency ultrasound probe at our disposal
- ◯ We do not have a curved low-frequency ultrasound probe at our disposal
- ◯ I do not know if we have a curved low-frequency ultrasound probe at our disposal

*** 22. I think Gastric Point-of-care Ultrasound should be more present in basic resident training**

- ◯ Yes
- ◯ No
- ◯ Neutral

Thank you !

We really want to thank you for answering this survey. If you have any remarks or questions, please write it below

23. Please share any other comments you have below:
